# Antihyperuricemic Effect of Urolithin A in Cultured Hepatocytes and Model Mice

**DOI:** 10.3390/molecules25215136

**Published:** 2020-11-04

**Authors:** Shin-ichi Adachi, Kazunori Sasaki, Shinji Kondo, Wataru Komatsu, Fumiaki Yoshizawa, Hiroko Isoda, Kazumi Yagasaki

**Affiliations:** 1 Center for Bioscience Research and Education, Utsunomiya University, Utsunomiya 321-8505, Tochigi, Japan; kondo.shinji.ga@u.tsukuba.ac.jp (S.K.); yagasaki@cc.tuat.ac.jp (K.Y.); 2 Alliance for Research on the Mediterranean and North Africa (ARENA), University of Tsukuba, 1-1-1 Tennodai, Tsukuba 305-8572, Ibaraki, Japan; sasaki-kazu@aist.go.jp (K.S.); isoda.hiroko.ga@u.tsukuba.ac.jp (H.I.); 3 Interdisciplinary Research Center for Catalytic Chemistry, National Institute of Advanced Industrial Science and Technology (AIST), Tsukuba 305-8565, Ibaraki, Japan; 4 Laboratory of International Environmental Health, Dokkyo Medical University School of Medicine, Mibu 321-0293, Tochigi, Japan; wkomatsu@dokkyomed.ac.jp; 5 Faculty of Agriculture, Utsunomiya University, Utsunomiya 321-8505, Tochigi, Japan; fumiaki@cc.utsunomiya-u.ac.jp; 6 Faculty of Life and Environmental Sciences, University of Tsukuba, Tsukuba 305-8572, Ibaraki, Japan

**Keywords:** urolithin A, ellagic acid, AML12 hepatocyte, hyperuricemia, uric acid

## Abstract

Hyperuricemia is defined as a disease with high uric acid (UA) levels in the blood and a strong risk factor for gout. Urolithin A (UroA) is a main microbial metabolite derived from ellagic acid (EA), which occurs in strawberries and pomegranates. In this study, we evaluated antihyperuricemic effect of UroA in both cultured hepatocytes and hyperuricemic model mice. In cultured hepatocytes, UroA significantly and dose-dependently reduced UA production. In model mice with purine bodies-induced hyperuricemia, oral administration of UroA significantly inhibited the increase in plasma UA levels and hepatic xanthine oxidase (XO) activity. In addition, DNA microarray results exhibited that UroA, as well as allopurinol, a strong XO inhibitor, induced downregulation of the expression of genes associated with hepatic purine metabolism. Thus, hypouricemic effect of UroA could be, at least partly, attributed to inhibition of purine metabolism and UA production by suppressing XO activity in the liver. These results indicate UroA possesses a potent antihyperuricemic effect and it could be a potential candidate for a molecule capable of preventing and improving hyperuricemia and gout.

## 1. Introduction

Hyperuricemia is the state characterized by abnormally high blood uric acid (UA) levels and caused by overproduction of UA mainly in the liver and/or reduced excretion from the kidney [1]. Hyperuricemia is regarded as an important risk factor for gout and also thought to increase the risk of other symptoms such as metabolic syndrome and renal disorders [2,3,4]. High levels of consumption of purine-rich foods such as meats, seafood, and purine-rich vegetables are associated with an increased risk of gout [5]. UA is the terminal product of the metabolism of purine nucleotides in human. Xanthine oxidase (XO) in the liver is the key enzyme of UA production, and urate transporters including URAT1, GLUT9, and OAT1 in the kidney are the main transporters for the excretion of UA [1]. UA production-suppressive drugs (e.g., allopurinol and febuxostat, potent XO inhibitors) and uricosuric drugs (e.g., benzbromarone) are prescribed in the treatment of hyperuricemia and gouts. However, these drugs has some of undesirable side effects such as gastrointestinal, hepatic, and renal toxicity [6,7,8,9]. Therefore, the search for novel substances in foods and natural resources that suppress hyperuricemia is highly warranted.

Urolithins are gut microbiota-derived metabolites of ellagic acid (EA), which is present in strawberries and pomegranates and also the metabolite of ellagitannins in gut. Among urolithins, urolithin A (UroA) has been reported to be the most abundant metabolite in human [10] and have a variety of beneficial effects in vitro and animal studies including anti-inflammatory, antidiabetic, antiobesity, and antioxidant activity and the enhancement of muscular performance [11,12,13]. Urolithin B (UroB) has been reported to be the final product catabolized among urolithins and exhibit anti-inflammatory and antioxidant effects [14,15]. Many polyphenols like isorhamnetin and taxifolin are reportedly effective against hyperuricemic model mice [16,17]. Previous research has shown quercetin, one of polyphenols, decreases plasma UA concentration in pre-hyperuricemic humans [18]. However, antihyperuricemic effect of UroA is not up to now.

We have recently contrived new assay systems in vitro and in vivo in combination for screening antihyperuricemic compounds [19]. In brief, UA levels in balanced salt solution (BSS) increased dose-dependently and significantly by addition of guanosine and inosine as the UA precursors, while the UA levels in AML12 hepatocytes were constant. Thus, the extracellular (BSS) UA level was considered to be a simple index of UA production in the cells. So far, in order to search medicinal and natural compounds that have hypouricemic activity, their direct inhibitory effects on XO activity were widely measured in vitro [20,21,22]. Screening in an XO assay system has a possibility to overlook compounds that have their targets other than XO. Therefore, the assay system in cultured hepatocytes would be useful to search novel natural and food compounds including those to possess targets other than XO. Besides the in vitro assay system, we have also constructed in vivo model mice with purine bodies-induced hyperuricemia to which nucleotides guanosine-5’-monophosphate (GMP) and inosine-5’-monophosphate (IMP) in combination are given as UA precursors.

In the present study, to investigate whether UroA could improve hyperuricemia, we assessed inhibitory effect of UroA on UA production in AML12 hepatocytes and plasma UA levels, hepatic XO activity, and renal UA transporter protein and gene expression in hyperuricemic model mice induced by purine-bodies administration. Additionally, the expression of hepatic genes related to purine metabolism were examined by DNA microarray.

## 2. Results

### 2.1. Effects of Ellagic Acid, Urolithin A and B on Cell Viability, and Uric Acid Production in AML12 Cells

**Figure 1:**
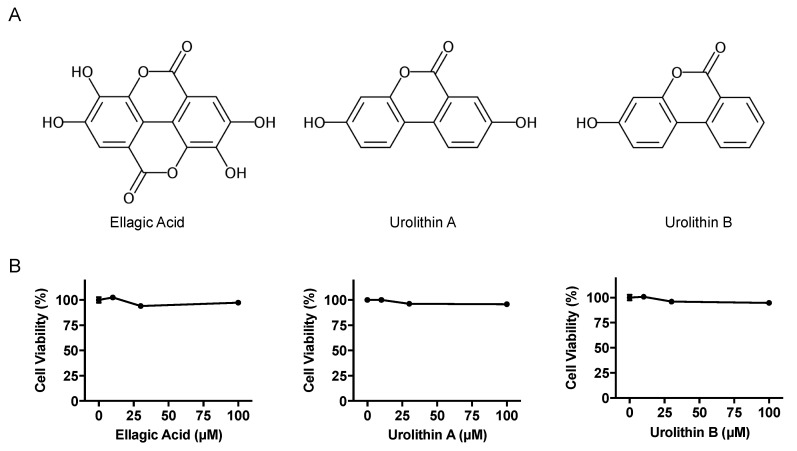
Chemical structures (**A**) and effects on cell viability (**B**) of ellagic acid, urolithin A, and urolithin B. Data are expressed as a percentage of the vehicle control (DMSO). Each value represents mean ± SEM for six wells.

**Figure 2:**
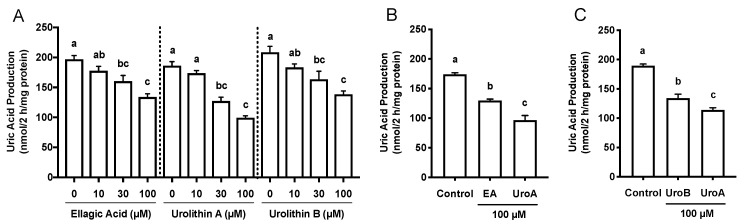
Effects of ellagic acid, urolithin A and urolithin B on UA production in AML12 cells (**A**). Comparison of effects of ellagic acid and urolithin A (**B**) and urolithin A and urolithin B (**C**) at the concentration of 100 µM on UA production in AML12 hepatocytes. EA, ellagic acid; UroA, urolithin A; UroB, urolithin B. Each value represents mean ± SEM for six wells (duplicate measurement per well). Values not sharing a common letter are significantly different at *p* < 0.05 (Tukey’s test).

The effects of EA, UroA, and UroB on the cell viability of AML12 hepatocytes were first assessed using WST-8 assay in this study. The treatment of AML12 cells with EA, UroA, and UroB did not show any cytotoxic effects up to the concentration of 100 µM (Figure 1B). Based on the consequences, we adopted the 100 µM of the compounds for evaluating the inhibitory effects on UA production in the hepatocytes without causing cellular damage. EA, UroA, and UroB dose-dependently and significantly reduced UA production in AML12 hepatocytes (Figure 2A). At the concentration of 100 µM, inhibitory effect of UroA was stronger than those of EA and UroB (Figure 2B,C). 

### 2.2. Effect of Ellagic Acid and Urolithin A and B on the Plasma Uric Acid Level in Hyperuricemic Model Mice

**Figure 3:**
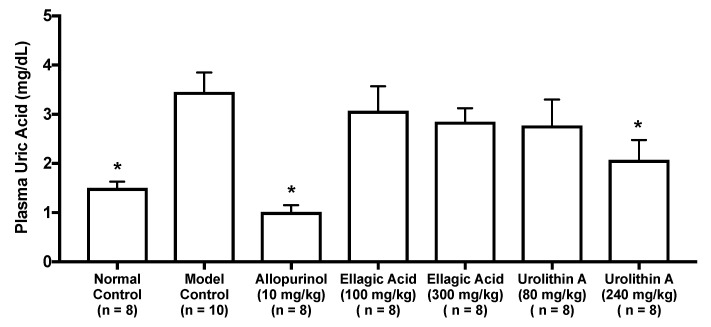
Effects of oral single-dose administration of ellagic acid and urolithin A on plasma UA levels in hyperuricemic model mice. The mice were perorally administered with ellagic acid or urolithin A at the different doses indicated. The mice were then intraperitoneally injected with both IMP and GMP (300 mg/kg body weight) to induce hyperuricemia. Normal control and model control groups were treated with 0.5% CMC-Na instead of test samples. Normal group was injected with PBS (–) instead of nucleotides. Each value represents mean ± SEM for 8–10 mice (duplicate measurement per mouse). For statistical significance, * *p* < 0.05 when the treated groups were compared with the model control group (Dunnett’s test).

**Figure 4:**
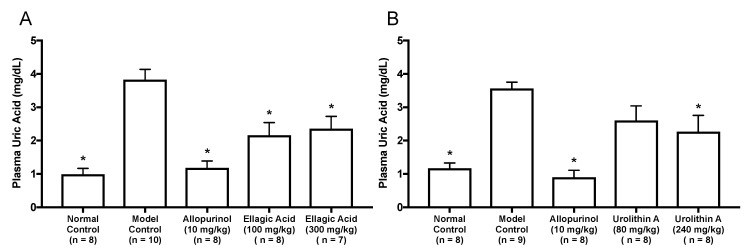
Effects of three-days oral administration of ellagic acid (**A**) and urolithin A (**B**) on plasma UA levels in hyperuricemic model mice. The mice were perorally administered with ellagic acid or urolithin A at the different doses indicated. The mice were then intraperitoneally injected with both IMP and GMP (300 mg/kg body weight) to induce hyperuricemia. Normal control and model control groups were treated with 0.5% CMC-Na instead of test samples. Normal group was injected with PBS (–) instead of nucleotides. Each value represents mean ± SEM for 7–10 mice (duplicate measurement per mouse). For statistical significance, * *p* < 0.05 when the treated groups were compared with the model control group (Dunnett’s test).

As in vitro antihyperuricemic effect of UroA was stronger than that of UroB (Figure 2C), we examined in vivo antihyperuricemic effect of UroA in purine bodies-induced hyperuricemic model mice. In single oral administration test, the intraperitoneal injection of GMP and IMP in combination into mice significantly increased the plasma UA level (model control group) than that of mice injected with PBS (–) alone (normal control group, Figure 3). This rise was significantly attenuated by allopurinol administration at 10 mg/kg BW. Similarly, UroA at 240 mg/kg BW also significantly suppressed the increase in plasma UA concentration as compared with that of model control group (Figure 3). In contrast, EA at 100 and 300 mg/kg BW and UroA at 80 mg/kg BW exerted no significant influence on the hyperuricemia. In three consecutive days of oral administration test, the plasma UA level significantly and strikingly increased in the model control group as compared with that in the normal control group (Figure 4A,B). This rise was significantly suppressed by allopurinol administration at 10 mg/kg body weight. Likewise, EA at 100 and 300 mg/kg BW and UroA at 80 and 240 mg/kg BW significantly cancelled the increase in plasma UA concentration as compared with that of model control group (Figure 4A,B).

### 2.3. Effect of Urolithin A on the Liver Xanthine Oxidase Activity in Hyperuricemic Model Mice

**Figure 5:**
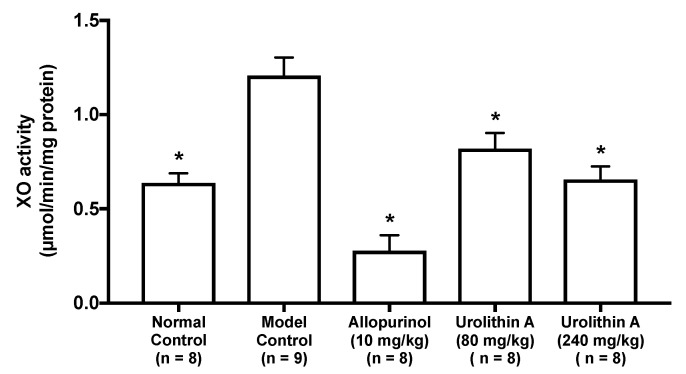
Effect of urolithin A on hepatic xanthine oxidase (XO) activity in hyperuricemic mice. Each value represents mean ± SEM for 8–9 mice (duplicate measurement per mouse). For statistical significance, * *p* < 0.05 when the treated groups were compared with the model control group (Dunnett’s test).

The intraperitoneal injection of GMP and IMP into mice significantly facilitated the hepatic XO activity than that of PBS (–) alone (normal control group vs. model control group, Figure 5). The XO activities in allopurinol (10 mg/kg body weight), low-dose (80 mg/kg BW) and high-dose (240 mg/kg BW) groups of UroA were significantly lower than that of model control group.

### 2.4. Gene Expression Profile of the Liver in Hyperuricemic Model Mice Treated with Urolithin A

The differences in hepatic gene expression between the model control and allopurinol (10 mg/kg BW) or high-dose groups (240 mg/kg BW) of UroA were comprehensively assessed by DNA microarray analysis. We focused on the differentially expressed genes related to UA production. As shown in Table 1, three genes related to UA production were significantly downregulated in the UroA-administered group compared to the model control group (Table 1). More specifically, UroA treatment induced the down-regulation of UA production-related genes, namely phosphoribosyl pyrophosphate synthetase 1-like 3 (Prps1l3), ectonucleoside triphosphate diphosphohydrolase 4 (Entpd4) and adenylate kinase 4 (Ak4) (*p* < 0.10; compared with the model control group). Interestingly, the UroA-administered group showed almost the same downregulation in these gene expression as the allopurinol (commercial UA production-suppressive drugs)-administered group.

### 2.5. Effects of Urolithin A on Kidney Uric Acid Transporter Protein in Hyperuricemic Mice

**Figure 6:**
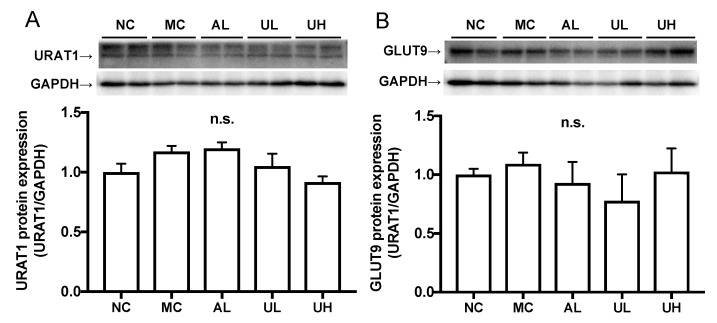
Effects of urolithin A on kidney URAT1 (**A**) and GLUT9 (**B**) protein expression in hyperuricemic mice by western blot analysis. NC, normal control; MC, model control; AL, allopurinol; UL, low-dose of urolithin A; UH, high-dose of urolithin A group. The bands were normalized to an internal control (GAPDH), presented as the relative ratio. Each value represents mean ± SEM for 4 mice.

There was no difference in the renal URAT1 and GLUT9 protein expression levels among normal control, model control, allopurinol, low-dose (80 mg/kg BW), and high-dose (240 mg/kg body BW) of UroA administration groups (Figure 6).

### 2.6. Effect of Urolithin A on Kidney Uric Acid Transporter Gene Expression in Hyperuricemic Mice

**Figure 7:**
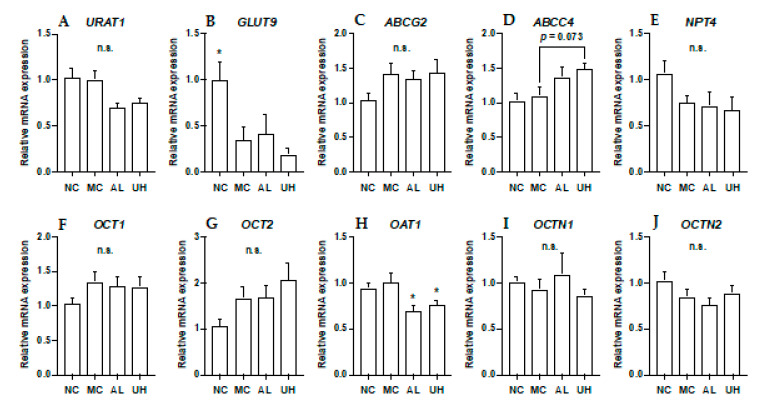
Effects of urolithin A on kidney mRNA expression of URAT1 (**A**), GLUT9 (**B**), ABCG2 (**C**), ABCC4 (**D**), NPT4 (**E**), OCT1 (**F**), OCT2 (**G**), OAT1 (**H**), OCTN (**I**), and OCTN2 (**J**). NC, normal control; MC, model control; AL, allopurinol; UH, high dose of urolithin A group. Each value represents mean ± SEM for 8 mice. For statistical significance, *p < 0.05 when the treated groups were compared with the model control group (Dunnett’s test).

The effects of allopurinol and UroA on mRNA levels of URAT1, GLUT9, ABCG2, ABCC4, NPT4, OCT1, OCT2, OAT1, OCTN1, and OCTN2 in hyperuricemic model mice were shown in Figure 7. The intraperitoneal injection of GMP and IMP into mice significantly down-regulated expression of renal GLUT9 in hyperuricemic model mice (Figure 7B). Allopurinol and UroA significantly downregulated expression of renal OAT1 in the mice (Figure 7H). Additionally, UroA tended to upregulate expression of renal ABCC4 in the model mice (*p* = 0.073, Figure 7D). There was no difference in the renal URAT1 ABCG2, NPT4, OCT1, OCT2, OCTN1, and OCTN2 mRNA expression levels among normal control, model control, allopurinol, and high-dose of UroA administration groups (Figure 7).

## 3. Discussion

Urolithins are dibenzopyran-6-one derivatives produced by gut microbiota from ellagitannins and EA. Ellagitannins has been found in a wide variety of fruits and nuts, such as pomegranate, strawberry, raspberry, walnuts, and muscadine grapes [10]. EA is generated from ellagitannins in gut, and plant-derived EA occurs in berries, grapes, and nuts [26]. Among the urolithins, UroA (3,8-dihydroxybenzo[*c*]chromen-6-one, Figure 1A) is reportedly the most abundant metabolite in human. UroB (3-Hydroxybenzo[*c*]chromen-6-one) has been reported to be the terminal product catabolized among the urolithins. In addition, it is known that ellagitannins and EA absorption is very low and that the unabsorbed compounds are further metabolized to urolithins by the gut microbiota [10]. Therefore, in this study, we have mainly focused on antihyperuricemic effect of UroA in cultured hepatocytes and mice with purine bodies-induced hyperuricemia.

In the recent study, UA precursors, i.e., guanosine and inosine, dose-dependently and significantly increased UA levels in BSS, but not within AML12 hepatocytes, which indicated that the extracellular UA level was considered to be a simple index for UA productivity [19]. In the present study, we first examined inhibitory effects of EA, UroA, and UroB on UA production by the cultured AML12 cells. They dose-dependently and significantly decreased UA production in the hepatocytes (Figure 2A). In the previous report, we examined the effect of allopurinol, a known XO inhibitor prescribed clinically for the treatment of hyperuricemia and gout, as the positive control drug on UA production in AML12 cells [16,19]. Allopurinol clearly and dose-dependently decreased UA production, demonstrating that the assay system worked precisely. At the identical concentration (100 µM), the effect of UroA was significantly stronger than EA and UroB (Figure 2B,C). From the high abundance ratio of UroA in the ellagitannins- and EA-metabolites and present results, we decided to select UroA but not UroB for the animal experiments. As a matter of course, hypouricemic effect of UroB on animals should be clarified in the future.

In both one day and three consecutive days oral administration test, UroA at dose of 240 mg/kg BW, as well as allopurinol for the positive control drugs in the present study, significantly cancelled the rise in plasma UA levels in purine bodies-induced hyperuricemia (Figure 3 and Figure 4B). These results provide evidence that UroA has an antihyperuricemic potential for the first time. Similarly, three consecutive days administration of EA at dose of 100 and 300 mg/kg BW significantly inhibited the rise in plasma UA levels (Figure 4A). On the other hands, in one day administration test, the rise was repressed by UroA at dose of 240 mg/kg BW but not by EA at dose of 100 and 300 mg/kg BW (Figure 3). UroA at 240 mg is almost equivalent molecular weight to EA at 300 mg. As above-mentioned, absorption of EA into intestinal tract is known to be very low, and unabsorbed EA is reported to be metabolized into urolithins by the gut microbiota. Therefore, in the animal study, a part of EA administrated on day 1 and 2 in the three consecutive days test might be metabolized to urolithins including UroA, and the EA-deprived UroA might suppress significantly the hyperuricemia although EA inhibited UA production in vitro assay. Further studies need to dissect the mechanism by which EA provides the benefit. In three consecutive days administration test, treatment with EA at dose of 100 mg/kg lowered the plasma UA level, but UroA at dose of 80 mg/kg was not significantly decreased the level. UroA has been reported to be metabolized into UroA glucuronides, which show weak bioactivity, and are excreted from the kidney [27]. Therefore, this inconsistency might be due to the reason that the bulk of UroA administrated on day 1 and 2 was metabolized into its glucuronides and/or excreted from the body. In contrast with low-dose of UroA group (80 mg/kg BW), in the low-dose of EA group (100 mg/kg BW), a part of EA might be metabolized into urolithins and the EA-deprived urolithins might remain in the mice with a time lag until the test on day 3. Further intensive studies are required to explain the reason precisely.

**Figure 8:**
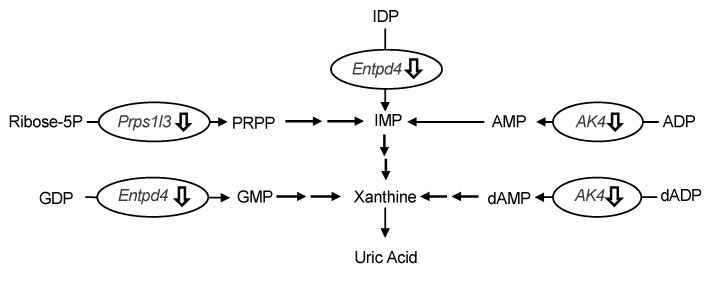
Effects of induction of administration of allopurinol and urolithin A on the gene expression of purine metabolism-related gene in the liver. Ribose-5P, ribose 5-phosphate; IMP, inosine-5’-monophosphate; IDP, inosine-5’-diphosphate; AMP, adenosine-5’-monophosphate; ADP, adenosine-5’-diphosphate; dAMP, deoxyadenosine-5’-monophosphate; dADP, deoxyadenosine-5’-diphosphate; GMP, guanosine-5’-monophosphate; GDP, guanosine-5’-diphosphate; Prps1l3, phosphoribosyl pyrophosphate synthetase 1-like 3; Entpd4, ectonucleoside triphosphate diphosphohydrolase 4; Ak4, adenylate kinase 4. Effects of induction of administration of allopurinol and urolithin A on the gene expression of purine metabolism-related gene in the liver. Ribose-5P, ribose 5-phosphate; IMP, inosine-5’-monophosphate; IDP, inosine-5’-diphosphate; AMP, adenosine-5’-monophosphate; ADP, adenosine-5’-diphosphate; dAMP, deoxyadenosine-5’-monophosphate; dADP, deoxyadenosine-5’-diphosphate; GMP, guanosine-5’-monophosphate; GDP, guanosine-5’-diphosphate; Prps1l3, phosphoribosyl pyrophosphate synthetase 1-like 3; Entpd4, ectonucleoside triphosphate diphosphohydrolase 4; Ak4, adenylate kinase 4.

The oral administration of UroA for three consecutive days decreased hepatic XO activity (Figure 5), indicating that the hypouricemic effect could be, at least partly, attributable to inhibition of UA production by directly suppressing XO activity in the liver. To ascertain the mechanism of decreased UA production in the liver, we evaluated the differences in hepatic gene expression of purine metabolism by using the DNA microarray technique. In this study, we identified three genes related to UA production in the liver (Table 1). Prps1l3, Entpd4, and Ak4 were downregulated by administration of allopurinol at 10 mg/kg or UroA at 240 mg/kg BW. It has been reported that Prps1l3 converts ribose 5-phospahte into phosphoribosyl pyrophosphate, precursor of IMP, that Entpd4 catalyzes hydrolysis of inosine-5’-diphosphate (IDP) and guanosine-5’-diphosphate (GDP) to IMP and GMP, respectively, and that AK4 catalyzes the reversible transfer of the terminal phosphate group between ATP and AMP [23,24,25]. Our data suggest that allopurinol and UroA may downregulate these genes, resulting in inhibition of purine metabolism and UA production (Figure 8). 

The kidneys play an important role in UA excretion. Morin, one of the flavonols, and green tea-derived polyphenols demonstrated hypouricemic effect by promotion of UA uptake from blood into urine via kidney and suppression of its reabsorption from urine into blood in the kidney of the potassium oxonate-induced hyperuricemic model mice [28,29]. Urate transporter 1 (URAT1) and glucose transporter 9 (GLUT9) are major players in the reabsorption of UA into blood, while ATP-binding cassette sub-family G member 2 (ABCG2), ATP-binding cassette sub-family C member 4 (ABCC4), Na^+^-dependent phosphate transporter 4 (NPT4), organic cation transporter 1 and 2 (OCT1 and OCT2), organic cation/carnitine transporter 1 and 2 (OCTN1 and OCTN2), and organic anion transporter 1 (OAT1) are involved in the secretion of UA into urine [1,30]. In the present study, allopurinol and UroA had no effect on URAT1 and GLUT9 protein expression levels in the kidney (Figure 6). In addition, they also had no effect on renal mRNA expression levels of URAT1, GLUT9, ABCG2, NPT4, OCT1, OCT2, OCTN1, and OCTN2 (Figure 7). On the other hands, mRNA level of ABCC4 tended to be increased by UroA administration (Figure 7D). This result indicate UroA may induce the uricosuric effect partially by upregulation of renal mABCC4 to promote UA secretion in the hyperuricemic model mice (Figure 7H). In addition, mRNA level of OAT1 was decreased by treatment with allopurinol or UroA. The decreased levels of OAT1 may be a homeostatic response to the decrease in plasma UA levels by allopurinol and UroA. OAT1 is estimated to transport UA from the blood into the proximal tubule cells as the first step of UA secretion [31]. Thus, mRNA level of OAT1 was first downregulated, and the levels of the other proteins involved in UA secretion, i.e., ABCG2, NPT4, OCT1, and OCTN1 might not receive any regulation. Furthermore, GMP and IMP significantly decreased mRNA level of GLUT9 (Figure 7B). The decreased levels of GLUT9 could be a homeostatic response to the increase in plasma UA levels by administration of GMP and IMP. Further studies should elucidate the mechanism by which UroA exhibits the uricouric actions in the kidney.

As above-mentioned, urolithins including UroA have good bioavailability and are present in plasma and urine at micromolar concentrations [32]. After absorption, urolithins rapidly undergo phase II metabolism, and are mostly metabolized into conjugated derivatives. The abundant metabolite of UroA has been reported to UroA glucuronide, which has poor bioactivity [27]. In rat model of lipopolysaccharide (LPS)-induced systemic inflammation, LPS and UroA treatment has been reported to elevate an activity of β-glucuronidase, an enzyme that catalyzes the hydrolysis of a glucuronide moiety from a variety of substrates, resulting in an increase in hepatic and renal UroA levels [33]. Additionally, the macrophage-mediated deconjugation of quercetin-glucuronide into quercetin was reported to be enhanced upon inflammatory activation by LPS in macrophage cells in the human atherosclerotic lesions [34]. Furthermore, we have reported that quercetin decreased hepatic and plasma UA levels in hyperuricemic model mice induced by purine-bodies administration [17]. Thus, these reports demonstrate that aglycones tend to accumulate in tissues or cells in sites of inflammation, and then exert their bioactivity. Although hepatic and renal inflammation induced by hyperuricemia remains unclear in detail, UroA is expected to be deconjugated from UroA glucuronide and exhibit antihyperuricemic action in sites of inflammation similarly to LPS models.

## 4. Materials and Methods

### 4.1. Materials

AML12 cells were provided by American Type Culture Collection (ATCC^®^ CRL2254, Manassas, VA, USA). DMEM/F-12 was purchased from Life Technologies (Grand Island, New York, NY, USA), fetal bovine serum (FBS) from Hyclone (Logan, UT, USA), penicillin and streptomycin from Nacalai Tesque, Inc. (Kyoto, Japan), Pierce^TM^ BCA Protein Assay kit from Thermo Fisher Scientific Inc. (Waltham, MA, USA). Urolithin A (UroA), guanosine-5’-monophosphate (GMP), and inosine-5’-monophosphate (IMP) were purchased from Tokyo Chemical Industry Co., Ltd. (Tokyo, Japan). Urolithin B (UroB), selenium, guanosine and inosine were purchased from Sigma-Aldrich Chemical Co. (St. Louis, MO, USA). Ellagic acid (EA), allopurinol, dexamethasone, carboxylmethyl cellulose sodium (CMC-Na), dimethylsulfoxide (DMSO), recombinant human insulin, transferrin from human blood, uric acid, and uric acid assay kit (Uric acid C-test Wako) were obtained from Wako Pure Chemical Industries, Ltd. (Osaka, Japan). Cell counting kit-8 was provided by Dojindo Laboratories (Kumamoto, Japan). The anti-GAPDH antibody were obtained from Santa Cruz Biotechnology, Inc., anti-URAT1 antibody from Thermo Fisher Scientific Inc., anti-GLUT9 antibody from Novus Biotechnology (Littleton, CO, USA), horseradish peroxidase (HRP)-conjugated anti-mouse and anti-rabbit IgG antibody from GE Healthcare (Chicago, IL, USA). The other regents were purchased from Wako Pure Chemical Industries, Ltd., and they were of guaranteed reagent grade.

### 4.2. Determination of Uric Acid Productions by AML12 Cells

AML12 cells were cultured in DMEM/F-12 supplemented with 10% FBS, 5 µg/mL recombinant human insulin, 5 µg/mL transferrin from human blood, 3 ng/mL selenium, 40 ng/mL dexamethasone, 100 U/mL penicillin, and 100 µg/mL streptomycin (10% FBS/DMEM/F-12) under an atmosphere of 5% CO_2_/95% humidified air at 37 °C as described previously [35] with slight modifications. The cells (1.0 × 10^5^ cells/well) into 24-place multiwell plates and grown for 72 h in 10% FBS/DMEM/F-12, and then kept for 24 h in serum-free DMEM/F-12. UA production by AML12 hepatocytes was evaluated as described previously [19]. In brief, after 24 h culture in serum-free DMEM/F-12, AML12 hepatocytes were washed once with phosphate buffered saline without calcium or magnesium (PBS (–)) and incubated in balanced salt solution (BSS) including 188 mM NaCl, 5 mM KCl, 1 mM MgCl_2_, 0.8 mM CaCl_2_, 25 mM NaHCO_3_, 1 mM NaH_2_PO_4_, 10 mM HEPES, and 5 mM glucose [35]. Furthermore, BSS contained guanosine and inosine (100 µM each) in combination (GI mixture) as UA precursors in the absence or presence of EA, UroA, and UroB (0, 10, 30, 100 µM) at the final DMSO concentration of 0.15%. On the termination of 2 h incubation, BSS was collected for determination of UA. UA detected in BSS was considered to be an index for UA productivity [19]. The hepatocytes were washed once with PBS (–) and scraped into buffer including 50 mM Tris and 1 mM sodium phosphate (pH 7.5). After being sonicated and centrifuged (12,000× *g*, 5 min, 4 °C), the supernatants were subjected to protein determination with a Pierce^TM^ BCA Protein Assay kit. UA levels in the BSS were determined by the uricase methods (uric acid C-test Wako). UA production was expressed as nmol per 2 h per mg cellular protein (nmol/2 h/mg protein).

### 4.3. Cell Viability Assay

Cell viability was determined by the Cell Counting Kit-8 according to the manufacture’s protocol with minor modifications [17]. AML12 cells were plated in 96-place multiwell plates at a density of 5 × 10^3^ cells per well and incubated for 72 h in 10% FBS/DMEM/F-12, and then kept for 24 h in serum-free DMEM/F-12. After 24 h, the 4.4hepatocytes were washed once with BBS and incubated in GI mixture in the absence or presence of EA, UroA, or UroB (0, 10, 30, 100 µM) for 2 h. After washed once with BSS, the cells were incubated in WST-8 reagent for 1 h. The optical density at 450 nm was read with Spark 10M (Tecan Group Ltd., Männedorf, Switzerland). The cell viability was expressed as a percentage of the value of cells treated without samples (DMSO control) as 100%.

### 4.4. Experimental Animals

Male ICR mice (Charles River Japan, Inc., Yokohama, Japan) at 4 weeks of age were housed in plastic cages in a room with a 12-h light/dark cycle (dark phase of 18:00–06:00) and constant temperature (22 °C). They were housed in groups of four mice for 7 days to acclimatize to the environment. The mice were maintained on tap water and regular diet (CRF-1, Oriental Yeast Co., Tokyo, Japan) ad libitum. This experiment was carried out in accordance with the guideline for Animal Experiments of Utsunomiya University Animal Research Committee (ethic approval number: A14-0017).

### 4.5. Urolithin A Administration to Hyperuricemic Model Mice

We evaluated antihyperuricemic effect of UroA on mice with purine-induced hyperuricemia by oral administration of UroA once a day for 1 day and 3 consecutive days as described previously [19]. In brief, after acclimatization to the environment for 1 week, the mice were divided into seven groups with similar body weight: normal group (*n* = 8), hyperuricemic model group (*n* = 10), allopurinol group (*n* = 8), low-dose of EA group (*n* = 8), high-dose of EA group (*n* = 8), low-dose of UroA group (*n* = 8), and high-dose of UroA group (*n* = 8). EA, UroA, and allopurinol were suspended in 0.5% CMC-Na. In a one day administration test, after 4 h fasting, allopurinol at 10 mg/kg body weight (BW), EA at 100 mg/kg (low-dose group) and 300 mg/kg BW (high-dose group) and UroA at 80 mg/kg (low-dose group) and 240 mg/kg BW (high-dose group) were orally given to the mice. UroA at 80 mg is almost equivalent molecular weight to EA at 100 mg. Mice of normal control and hyperuricemic model control group were orally given 0.5% CMC-Na alone. The mice were intraperitoneally injected with both GMP and IMP (300 mg each/kg BW) to induce hyperuricemia 1 h after allopurinol, EA, UroA, or the vehicle (the model control). GMP and IMP were dissolved in PBS (–). The normal control group was injected with the PBS (–) alone as a vehicle. One hour after GMP and IMP injection, the blood was collected under isoflurane anesthesia from the inferior vena cava in the microtube with heparin sodium. For three consecutive days of oral administration test of EA, the mice were divided into five groups with similar body weight: normal group (*n* = 8), hyperuricemic model group (*n* = 10), allopurinol group (*n* = 8), low-dose of EA group (*n* = 8), and high-dose of EA group (*n* = 7). For three consecutive days oral administration test of UroA, the mice were divided into five groups with similar body weight: normal group (*n* = 8), hyperuricemic model group (*n* = 9), allopurinol group (*n* = 8), low-dose of UroA group (*n* = 8) and high-dose of UroA group (*n* = 8). Allopurinol at 10 mg/kg BW, EA at 100 mg/kg and 300 mg/kg BW and UroA at 80 mg/kg and 240 mg/kg BW were orally given to the mice once a day for the three consecutive days. On day 3, the mice were intraperitoneally injected with both GMP and IMP (300 mg each/kg BW) 1 h after the samples or the vehicle. One hour after GMP and IMP injection, the blood was collected and the liver and kidney were excised. The blood samples were centrifuged at 5000× *g* for 10 min at 4 °C to obtain the plasma. The plasma was stored at −80 °C until analyzed. The excised liver and kidney were washed with saline, cut into two pieces, frozen in liquid nitrogen, and stored at −80 °C until analyzed [16]. Plasma UA levels were measured by the uricase method (uric acid C-test Wako).

### 4.6. Liver Xanthine Oxidase Activity Assay

Liver XO activity assay was performed according to the procedure as previously described [36] with slight modifications using 96-well multi-plates [16]. The liver sample was homogenated in ice-cold 100 mM Tris-HCl (pH 7.5) containing 1 mM EDTA-Na, sonicated, and centrifuged (10,000×*g*, 5 min, 4 °C). The supernatant fraction was used to determine XO activity. Liver homogenates (40 µL) and ice-cold 100 mM Tris-HCl (pH 7.5) containing 1 mM EDTA-2Na (30 µL) were applied into 96-well plates. The reaction was initiated by the addition of 180 µl of 150 µM xanthine in the same buffer. Immediately after the addition of the substrate buffer, the absorbance at 295 nm and 37 °C was measured with Spark 10M for 30 min. UA production was calculated from the increase of the absorbance for 30 min based on the UA standard curve. Protein concentrations in the liver homogenates were determined with Pierce^TM^ BCA Protein Assay kit. XO activity was expressed as µmol UA produced per min mg protein.

### 4.7. DNA Microarray Analysis

In order to examine the differentially expressed genes in the livers among the normal, hyperuricemic model, allopurinol, and high-dose UroA groups, we conducted DNA microarray analysis. In each group, we selected three mice that had plasma uric acid levels close to the mean value of the group. The total RNA was extracted from the liver sample using the ISOGEN kit (Nippon Gene Co. Ltd., Toyama, Japan), as previously described [37]. Total RNA was quantified and assessed for its quality with NanoDrop 2000 spectrophotometer (Thermo Scientific, Wilmington, DE, USA). DNA microarray analysis was performed as reported previously [38,39]. In brief, double-stranded cDNA was synthesized from 100 ng of total RNA with the GeneAtlas 3′ IVT Express Kit (Affymetrix Inc., Santa Clara, CA, USA). Biotin-labeled amplified RNA (aRNA) was synthesized by in vitro transcription using the GeneChip 3′ IVT Express Kit (Affymetrix Inc.). A total of 9.4 mg of purified aRNA was fragmented using the GeneAtlas 3′ IVT Express Kit and was hybridized for 16 h at 45 °C using GeneChip MG-430 PM microarray (Affymetrix Inc.). The chip was washed and stained in the Gene Atlas Fluidics Station 400 (Affymetrix Inc.) and then the resulting image was scanned using the GeneAtlas Imaging Station (Affymetrix Inc.). Data analysis was performed using the Partek Express software (Partek Inc., St. Louis, MO, USA) provided by Affymetrix as part of their GeneAtlas system. The data was normalized using the Affymetrix expression console (http://www.affymetrix.com). Compared with the model control, fold change in expression in the allopurinol or high-dose of UroA group was calculated and converted to log 2 data.

### 4.8. Western Blotting

Immunoblotting was carried out as previously described [40], with slight modifications [17]. In each group, four mice that had plasma uric acid levels close to the mean value of the group were selected. Kidney protein samples were separated by SDS-PAGE, transferred to a PVDF membrane which was blocked for 1 h with 5% bovine serum albumin in Tris-buffered saline with 0.1% Tween-20 (Sigma-Aldrich Chemical Co.), and incubated overnight at 4 °C with the primary antibody. The primary anti-body was detected with HRP-conjugated anti-mouse secondary antibody and visualized with Amersham^TM^ ECL^TM^ western blotting detection reagent.

### 4.9. Real-Time Quantitative PCR Analysis

Expression of target and reference genes in the kidney was monitored by quantitative real-time PCR with GAPDH used as reference, as previously described [41]. In brief, total RNA was isolated from kidneys in normal control, model control, allopurinol and high-dose of UroA groups according to the Trizol-chloroform protocol (Thermo Fisher Scientific). cDNA was synthesized from 1µg of total RNA using iScript reverse tanscriptase (Bio-rad, Hercules, CA, USA), and qRT-PCR was conducted by using the MyiQ2 real-time PCR System (Bio-rad). The details of primer sequences were referenced in the previous reports [42,43,44]. Primer sequences were as follows; *URAT1* forward, GCTACCAGAATCGGCACGCT; *URAT1* reverse, CACCGGGAAGTCCACAATCC; *GLUT9* forward, GAGATGCTCATTGTGGGACG; *GLUT9* reverse, GTGCTACTTCGTCCTCGGT; *ABCG2* forward, TAAATGGAGCACCTCAACCT; *ABCG2* reverse, GAGATGCCACGGATAAACTG; *ABCC4* forward, TAATGGAAGCAGACAAGGCCCAGA; *ABCC4* reverse, AGAGGCCAGTGCAGATACATGGTT; *NPT4* forward, TCTGCACCATTGCCTTGTCA; *NPT4* reverse, CAAATACCCATCTAGACAACACATCTTT; *OCT1* forward, ACATCCATGTTGCTCTTTGG; *OCT1* reverse, TTGCTCCATTATCCTTACCG; *OCT2* forward, ACAGGTTTGGGCGGAAGT; *OCT2* reverse, CACCAGAAATAGAGCAGGAAG; *OAT1* forward, GCCTATGTGGGCACCTTGAT; *OAT1* reverse, CTTGTTTCCCGTTGATGCGG; *OCTN1* forward, AGGAGAGGTGGAAACATGCG; *OCTN1* reverse, TCCTTCGTCTCCAAGGGGAT; *OCTN2* forward, CTTATTCCCATACGGGCGCT; *OCTN2* reverse, TTTCTGAGGCACCTGTCGTC; *GAPDH* forward, TGAGGCCGGTGCTGAGTATGT; *GAPDH* reverse, CAGTCTTCTGGGTGGCAGTGAT.

### 4.10. Statistical Analysis

Data are expressed as means ± SEM. Data on cell viability and UA production in AML12 hepatocytes were analyzed by one-way ANOVA and Tukey’s multiple-comparisons test as a post hoc test. The results of the animal experiment except for DNA microarray were analyzed by one-way ANOVA with Dunnett’s multiple-comparisons test. Statistical analysis in microarray was performed based on unpaired two-tailed Student’s *t*-test. *p* values < 0.05 were considered statistically significant except for DNA microarray study. *p* values < 0.1 were considered statistically significant in DNA microarray study. These analyses were conducted by using the Prism 6 software package (GraphPad, San Diego, CA, USA).

## 5. Conclusions

In the present study, we have demonstrated that UroA, one of the metabolites of EA, suppresses UA production in cultured hepatocytes and inhibits the increase of the plasma UA levels in mice with hyperuricemia induced by purine bodies, at least partly, due to its effect on hepatic XO activity. In addition, antihyperuricemic effect of UroA on the model mice also appear to be due to inhibition of purine metabolism and UA production in the liver. Although further studies are required to elucidate precise mechanisms involved, UroA may be a promising agent for hyperuricemia-treatment.

## Figures and Tables

**Table 1:** List of purine metabolism-related genes among differently expressed genes in the liver and their differences between model control and allopurinol groups or model control and urolithin A high dose groups.

Gene Symbol	Gene Name	Fold Change (MC vs. AL)	Fold Change (MC vs. UH)	Molecular Function
Prps1l3	phosphoribosyl pyrophosphate synthetase 1-like 3	0.73 *	0.70 *	Converts ribose 5-phosphate into phosphoribosyl pyrophosphate (PRPP) [23]
Entpd4	ectonucleoside triphosphate diphosphohydrolase 4	0.84 **	0.76 **	Catalyzes the hydrolysis of nucleotide diphosphates and triphosphates in a calcium or magnesium-dependent manner [24]
Ak4	adenylate kinase 4	0.78 **	0.76 **	Catalyze the reversible transfer of the terminal phosphate group between ATP and AMP [25]

Table values are expressed as mean ± SEM for three mice in each group. * *p* < 0.10, ** *p* < 0.05 in comparison to model control mice.
